# Prevalence risk of sarcopenia in older Brazilian adults during the pandemic: A cross-sectional analysis of the Remobilize Study

**DOI:** 10.1590/1516-3180.2022.0159.R1.19082022

**Published:** 2022-12-19

**Authors:** Patricia Parreira Batista, Monica Rodrigues Perracini, Juleimar Soares Coelho de Amorim, Maria do Carmo Correia de Lima, Camila Astolphi Lima, Daniele Sirineu Pereira, Renata Gonçalves Dantas, Etiene Oliveira da Silva Fittipaldi, Aurélio Dias Santos, Hércules Lázaro Morais Campos, Leani Souza Máximo Pereira

**Affiliations:** I PT, MSc. Doctoral Student Postgraduate Program in Rehabilitation Sciences, Department of Physiotherapy, Universidade Federal de Minas Gerais (UFMG), Belo Horizonte (MG), Brazil.; II PT, PhD. Professor, Master’s and Doctoral Programs in Physical Therapy, Universidade Cidade de São Paulo (UNICID), São Paulo (SP), Brazil; Master’s and Doctoral Programs in Gerontology, Faculty of Medical Sciences, Universidade Estadual de Campinas (UNICAMP), Campinas (SP), Brazil.; III PT, PhD. Professor, Physical Therapy Course, Instituto Federal do Rio de Janeiro, Rio de Janeiro (RJ), Brazil.; IV PT, PhD. Faculty of Medical Sciences, Master’s and Doctoral Programs in Gerontology, Universidade Estadual de Campinas (UNICAMP), Campinas (SP), Brazil.; V PT, PhD. Postdoctoral Student of Master’s and Doctoral Program in Physical Therapy, Universidade Cidade de São Paulo (UNICID), São Paulo (SP), Brazil.; VI PT, PhD. Professor, Postgraduate Program in Rehabilitation Sciences, Department of Physiotherapy, Universidade Federal de Minas Gerais (UFMG), Belo Horizonte (MG), Brazil.; VII PT, MSc. Doctoral Student of Master’s and Doctoral Program in Physical Therapy, Universidade Cidade de São Paulo (UNICID), São Paulo (SP), Brazil; and Professor of Physical Therapy, Universidade Estadual do Sudoeste da Bahia (UESB), Vitória da Conquista (BA), Brazil.; VIII PT, PhD. Professor, Department of Physiotherapy, Universidade Federal de Pernambuco (UFPE), Recife (PE), Brazil.; IX PT, MSc. Professor, Department of Physiotherapy, Centro Universitário Dr. Leão Sampaio (UNILEÃO), Juazeiro do Norte (CE), Brazil.; X PT, MSc. Professor, Department of Physiotherapy, Universidade Federal do Amazonas/Instituto de Saúde e Biotecnologia (UFAM/ISB), Coari (AM), Brazil. Doctoral Student, Postgraduate Program in Public Health, Universidade Federal do Espírito Santo (UFES), Vitória (ES), Brazil.; XI PT, PhD. Professor, Postgraduate Program in Rehabilitation Sciences, Department of Physiotherapy, Universidade Federal de Minas Gerais (UFMG), Belo Horizonte (MG), Brazil.

**Keywords:** Sarcopenia, Mobility limitation, Aged, Physical distancing, Pandemics, SARC-F, Risk of sarcopenia, Screening, Older adults

## Abstract

**BACKGROUND:**

Social distancing has led to lifestyle changes among older adults during the coronavirus disease 2019 (COVID-19) pandemic.

**OBJECTIVES:**

This study aimed to estimate the prevalence risk of sarcopenia (RS) and investigate its associated factors during the COVID-19 pandemic in older Brazilian adults.

**DESIGN AND SETTING:**

Cross-sectional observational analysis of baseline data as part of the Remobilize Study.

**METHODS:**

Participants in the study were older adults (≥ 60 years), excluding those who were bedridden or institutionalized. The data collected consisted of answers about the RS (SARC-F), functional status, walking, sedentary behavior (SB), pain, comorbidity, and life space mobility.

**RESULTS:**

A total of 1,482 older adults (70 ± 8.14 years, 74% women) participated in the study, and an RS prevalence of 17.1% was found. (95% confidence interval [CI] 15.25–19.15%). The adjusted multivariate model showed a significant association between RS and functional limitation (odds ratio [OR]: 19.05; CI 13.00–28.32), comorbidity (OR: 5.11; CI 3.44–7.81), pain (OR: 4.56; CI 3.33–6.28), total walking (OR: 0.99; CI 0.99–1.00), SB of 8–10 hours (OR: 1.85; CI 1.15–2.93), and SB of > 10 hours (OR: 3.93; CI 2.48–6.22). RS was associated with mobility during the pandemic (OR: 0.97; CI 0.96–0.98). P *<* 0.05.

**CONCLUSIONS:**

During the pandemic, the prevalence of RS in older Brazilians was estimated at 17.1%. Moderate to severe functional limitation, comorbidities, presence of pain, walking, longer SB period, and reduced life space mobility significantly contributed to RS in older adults during the pandemic.

## INTRODUCTION

Social restriction policies and lifestyle changes favor a reduction in mobility and the level of physical activity (PA), leading to a higher proportion of inactive people and an increase in sedentary behaviors (SB) during the pandemic.^1-4^ A decline in life space mobility contributes to a reduction in intrinsic capacity, higher risk of sarcopenia (RS), and other adverse health consequences.^5,6^ After 7 days of total bed rest, there is already a significant deterioration in muscle function in community-dwelling older adults, and 2,000 steps per day are not enough to prevent these deleterious effects on the musculature.^7^ Coker et al. reported that a 15-day bed rest induces a significant reduction in fat-free muscle mass, poor performance, and increased fat in older individuals, which negatively impacts their mobility.^8^

A longer SB time observed during the pandemic is related to a worse prognosis in health conditions and a higher RS.^1-3,7,9^ These factors can alter the homeostasis between the pro- and anti-inflammatory systemic components and muscle anabolism and catabolism, leading to the reduction of physiological reserves in older adults. Consequences such as increased plasma pro-inflammatory cytokines, greater muscle catabolism drive, and anabolic and insulin resistance lead to a deleterious cycle of muscle function, explaining the higher incidence of RS in this population.^1-3^

Sarcopenia is a generalized and progressive musculoskeletal disorder that is defined as a reduction in muscle mass and strength. It is a multifactorial disease with dynamic interrelationships and is commonly associated with a cascade of negative repercussions on health, functional limitation, and mortality.^10-12^ Consequently, due to its considerable clinical impact on older individuals, it increases health-related expenses and imposes a burden on the public health system, being more costly in socially unequal and/or developing countries, such as Brazil.^10,12,13^ Updates from the European Working Group on Sarcopenia in Older People (EWGSOP2) and the Asian Working Group for Sarcopenia proposed the practice of population screening for RS in older people through strength, assistance with walking, rising from a chair, climbing stairs, and falls (SARC-F) questionnaire, a self-reported screening questionnaire.^10,14^ Identifying sarcopenia in its early stages enables the control of its progression and/or reversal of the individual’s clinical condition, thereby reducing the negative impacts caused by the disease.^3,10,11,14,15^

## OBJECTIVE

Due to the abovementioned reasons, this study aimed to verify the prevalence of RS and investigate the factors associated with the presence of RS during the coronavirus disease 2019 (COVID-19) pandemic.

## METHODS

### Design and sample

This study presents a cross-sectional analysis of baseline data collected from May to July 2020 through an online questionnaire as part of the Remobilize Study (www.remobilize.com.br).^4^ Using convenience snowball sampling, the online questionnaire (SurveyMonkey platform) was distributed throughout the Brazilian territory via social media (Facebook and Instagram), WhatsApp groups, social groups for older adults, and/or their friends and acquaintances. A pilot project for calibration and adjustments was conducted in advance. This study was approved by the University City of São Paulo Research Ethics Committee (May 18, 2020; CAAE 31592220.6.0000.0064) and is currently under progress.

The sample population consisted of community-dwelling older Brazilians (≥ 60 years) without distinction of sex, race, and/or social class. Following the exclusion criteria, those residing in long-term care facilities and/or bedridden were not eligible to participate in the study.^4^ Participants who presented with disabilities were allowed to have the questions be answered by a family member or caregiver.^16^ Participants without familiarity with the Internet were able to answer the survey via telephone.^4^

### Measures

The sociodemographic, clinical, and lifestyle data are presented in Table 1. The self-reported functional comorbidity index questionnaire was used to detect the presence of comorbidities (two or more chronic diseases).^17^ All participants answered questions about the presence or absence of pain.

**Table 1.** Total sample descriptive data and comparison between the groups with (strength, assistance with walking, rising from a chair, climbing stairs, and falls, SARC-F ≥ 4 points) and without risk of sarcopenia (RS) (SARC-F < 4 points)

		SARC-F		P value
		< 4 points (n = 1,228)	≥ 4 points (n = 254)	
**Age, %**	60–69 years	61.3%	31.5%	< 0.0001
	70–79 years	28.8%	26.0%	
	80 years and older	9.9%	42.5%
**Sex, %**	Male	27.9%	16.9%	0.001
	Female	72.1%	83.1%	
**Marital status, %**	Single	10.3%	10.2%	< 0.0001
	Married	56.7%	39.0%	
	Divorced	12.7%	11.0%	
	Widowed	20.3%	39.8%	
**Education, %**	Illiterate	6.4%	14.9%	< 0.0001
	1–4 years	16.5%	31.5%	
	5–8 years	11.9%	13.8%	
	9 years or more	65.2%	39.8%	
**Income^a^, %**	Up to 1× minimum wage	32.6%	44.1%	< 0.0001
	2–3× minimum wage	27.9%	27.9%	
	4–7× minimum wage	19.4%	11%	
	8–10× minimum wage	7.6%	7.9%	
	More than 10× minimum wage	12.5%	9.1%
**Occupation, %**	Active	39.2%	24.8%	< 0.0001
	Inactive	55.3%	61.8%	
	Unemployed	5.5%	13.4%	
**Sitting time, %**	< 4 hours	48.6%	28.8%	< 0.0001
	5–7 hour	31.0%	31.1%	
	8–10 hour	12.6%	16.9%	
	> 10 hours	7.8%	23.2%	
**BOMFAQ (4 pts +), %**		10.3%	73.6%	< 0.0001
**Comorbidities (≥ 2), %**		50.40%	87.40%	< 0.0001
**Pain (yes), %**		21.6%	55.5%	< 0.0001
**Walking (exercise)** **Med (IQR)**		0 (0–25.31)	0 (0–0)	< 0.0001
**Walking (utilitarian)** **Med (IQR)**		7.5 (0–33.75)	0 (0; 0)	< 0.0001
**Walking (total)** **Med (IQR)**		7.5 (0; 101.20)	0 (0; 7.5)	< 0.0001
**LSA - During pandemic** **Med (IQR)**	Total score	36 (24; 52)	24 (12; 32)	< 0.0001
	Level 1	8 (8; 8)	8 (6; 8)	< 0.0001
	Level 2	16 (12; 16)	12 (4; 16)	< 0.0001
	Level 3	6 (0; 12)	0 (0; 6)	< 0.0001
	Level 4	8 (0; 16)	0 (0; 4)	< 0.0001
	Level 5	0 (0; 0)	0 (0; 0)	0.0002

Med = median; IQR = interquartile range (1^st^ and 3^rd^ IQR); LSA = Life-Space Assessment; BOMFAQ = Brazilian OARS Multidimensional Functional Assessment Questionnaire; a score of four points or more refers to the presence of moderate to severe functional limitation; walking (as exercise, utilitarian, and total) in the previous week (min/week). ^a^minimum wage in Brazil = R$ 1,100.00 per month, corresponding to US$ 194.01 (April 5, 2021). The prevalence of total sarcopenia and per SARC-F item is reported as %. The prevalence of each SARC-F item refers to the sum of the two options (some difficulty or great difficulty). Study flowchart extracted from Perracini et al.^4^

The SARC-F questionnaire is recommended by the EWGSOP2 and the Asian Working Group for Sarcopenia as a population screening tool for RS.^10,14^ The final score ranges from zero to ten points, and a score of ≥ 4 points identifies individuals with sarcopenia. SARC-F has a high specificity, but low to moderate sensitivity.^10,14,15,18^ Population screening for RS (SARC-F) allows the exclusion of older patients with preserved muscle function in primary health care and identification of changes in the early stages of muscle function, functionality, and RS in older adults.^10,14,15,19^

Functional performance was assessed using the Older American Resources and Services questionnaire that has been translated and validated for the Brazilian population (BOMFAQ).^20,21^ It is a self-report questionnaire on the ability to perform 15 functional activities (eight basic and seven instrumental tasks). The scores for the activities performed with difficulty or requiring help were added, ranging from 0–15 points. Older adults were classified based on their scores: no (0), slight (1–3), moderate (4–6), and severe (> 7) functional limitation.^22^

SB was assessed using one question about the duration of sitting activities in the prior week, referring to indoor activities (≤ 4 hours/day, 5–7 hours/day, 8–10 hours/day or ≥ 10 hours/day). Walking, including PA, utilitarian walking, and walking time, was assessed using the Incidental and Planned Exercise Questionnaire.^23^ Validated for older adults, this is a simple, self-report questionnaire probing on walking activities during the prior week, specifically on the frequency and duration of the activity. The final score for walking as physical exercise and utilitarian walking was given by the product of frequency and duration for each item (minutes/week). The total walking time was calculated as the sum of walking as PA and utilitarian walking.

Life space mobility was measured using the Life-Space Assessment (LSA).^24^ It estimates the individual perspective of mobility relative to the spatial area in five levels of life space in the prior week: mobility in the rooms at home, outside the bedroom (level 1), outside the home (level 2), a neighborhood close to home (level 3), circulation within the municipality where they reside (level 4), and inter-municipal areas (level 5). The answers were based on the frequency and need for mobility devices. The score was calculated as the product of frequency and performance skill, extracting a score based on level and the total by the sum of levels (0–120 points). Higher final scores indicated better mobility performance in the life space.

### Statistical analysis

The prevalence of RS in participants was estimated using a 95% confidence interval (CI). Descriptive statistics were performed using absolute and relative frequencies for the total sample and RS, respectively. Continuous variables did not show a normal distribution in the Shapiro–Wilk test; therefore, the data are presented as medians and interquartile ranges. To compare the groups with and without RS, Pearson’s chi-square test was used for categorical variables and the Mann–Whitney test was used for continuous variables. The association between independent variables and outcome was based on odds ratios (ORs) estimates and their respective CIs through logistic regression without (crude model) and with adjustment (adjusted model). All analyses were performed using Stata 14.0 (StataCorp LLC, College Station, Texas, United States), with a 5% statistical significance level.

## RESULTS

**Figure 1.**
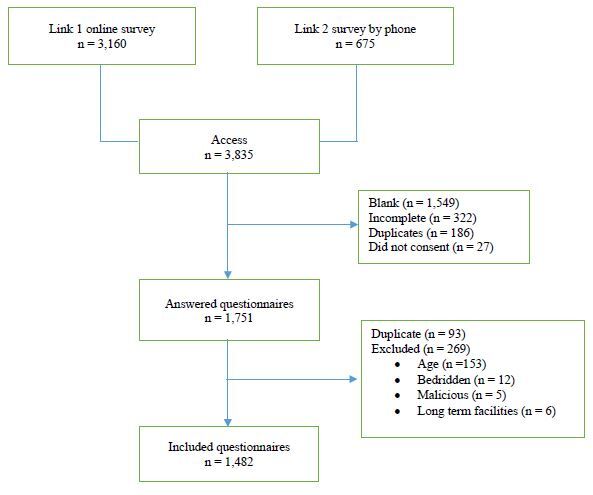
Study flowchart.

**Figure 2.**
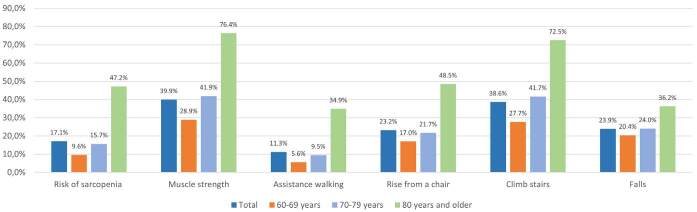
Prevalence of risk of sarcopenia in the total sample and by age group. Frequency of the items included in the strength, assistance with walking, rising from a chair, climbing stairs, and falls (SARC-F) questionnaire for older at risk for sarcopenia.

A total of 1,482 participants were included in this study, and the study flowchart is shown in Figure 1. The prevalence of RS during the pandemic was 17.1% (CI 15.25–19.15%). The distribution of SARC-F and total score items by age group and total scores are shown in Figure 2. Statistically significant differences were observed between the groups with and without RS in terms of age, sex, marital status, education, income, occupation, walking (exercise, utilitarian, and total), sitting time, functional limitation, presence of comorbidities, and pain. The RS group had a higher proportion of participants aged 80 years or older (42.5%), women (83.1%), lower income (44.1%), and 73.6% presented with moderate to severe functional limitation (Table 1). There were statistically significant differences in total LSA scores during the pandemic. Lower LSA scores were observed in older patients with RS. During the pandemic, there was a difference between older patients with and without RS for all walking variables (exercise and total), with lower values in the RS group.

Study flowchart extracted from Perracini et al.^4^

Crude logistic regression analysis showed a significant association for all analyzed variables. After adjusting for sociodemographic factors, the following variables remained statistically significant, as seen in Table 2: moderate to severe functional limitations, comorbidity, pain, walking (exercise and total), SB 8–10 hours, SB > 10 hours, and total LSA score during the pandemic.

**Table 2.** Logistic regression analysis to verify the association of the factors contributing to the risk of sarcopenia

		Crude model			Adjusted model		
		OR	95% CI	P value	OR	95% CI	P value
BOMFAQ (4 pts +)(ref.: 0–3 pts)		24.20	17.42; 33.40	< 0.001	19.05	13.00; 28.32	< 0.001
Comorbidities (≤ 2)(ref.: 0–1)		6.82	4.70; 10.22	< 0.001	5.11	3.44; 7.81	< 0.001
Pain(ref.: absence of)		4.54	3.42; 6.03	< 0.001	4.56	3.33; 6.28	< 0.001
Walking (exercise)		0.99	0.986; 0.994	< 0.001	0.99	0.989; 0.997	0.001
Walking (utilitarian)		0.99	0.994; 0.999	0.041	0.99	0.997; 1.001	0.346
Walking (total)		0.99	0.993; 0.997	< 0.001	0.99	0.995; 0.999	0.008
Sedentary behavior(sitting time; ref. < 4 hours)	5–7 hour	1.69	1.20; 2.39	0.003	1.41	0.97; 2.04	0.072
	8–10 hour	2.26	1.48; 3.42	< 0.001	1.85	1.15; 2.93	0.01
	> 10 hours	5.02	3.34; 7.53	< 0.001	3.93	2.48; 6.22	< 0.001
LSA - During pandemic	Total score	0.95	0.94; 0.96	< 0.001	0.97	0.96; 0.98	< 0.001
	Level 1	0.79	0.74; 0.84	< 0.001	0.83	0.77; 0.89	< 0.001
	Level 2	0.90	0.88; 0.92	< 0.001	0.92	0.90; 0.95	< 0.001
	Level 3	0.92	0.91; 0.94	< 0.001	0.95	0.93; 0.97	< 0.001
	Level 4	0.93	0.91; 0.95	< 0.001	0.97	0.95; 0.98	< 0.001
	Level 5	0.92	0.88; 0.96	< 0.001	0.96	0.91; 0.99	0.047

Med = median; IQT= interquartile range (1^st^ and 3^rd^ IQR); LSA = Life-Space Assessment; BOMFAQ = Brazilian OARS Multidimensional Functional Assessment Questionnaire; a score of four points or more refers to the presence of moderate to severe functional limitation. Walking (as exercise, utilitarian, and total) in the previous week (minutes/week).

## DISCUSSION

The results showed a high prevalence of RS in older Brazilians at the beginning of the COVID-19 pandemic in Brazil and a substantial association between RS and moderate to severe functional limitation, comorbidities, pain, and a positive gradient with the number of hours in SB. The OR for RS increased from 1.85 in older patients who reported 8 to 10 hours of SB to 3.93 in those with 10 hours or more of SB. Older patients with moderate to severe functional limitation were 19.05 times more likely to be at RS. Furthermore, greater mobility in living spaces lowered the chances of RS during the pandemic.

The prevalence of RS (17.1%) in the present study was substantially higher than that found in studies before the COVID-19 pandemic.^25-28^ Dodds et al. reported a 4% prevalence of RS in 1,686 British older adults (aged ≥ 69 years),^25^ while Kim and Won reported a rate of 7.5% among 2,123 Korean older adults (75.9 years).^26^ Studies with a model of activity reduction (steps per day) in elders pointed to negative repercussions of greater catabolic drive on their musculature and metabolic and inflammatory markers during a short period of mobility restriction.^29,30^ With a 76% reduction in steps per day (< 1,500 steps/day) in 14 days, Breen et al. demonstrated a 3.9% reduction in fat-free lean mass; reduced insulin sensitivity (43%); and increased pro-inflammatory cytokines, TNF-α (12%), and C-reactive protein (25%) levels in 10 healthy older adults after the intervention (72.3 years).^30^ These findings may support the higher RS prevalence in our study.

In Brazil, Barbosa-Silva et al. reported that sarcopenia had a prevalence of 8.4% (EWGSOP1) in 179 older adults.^18^ Sarcopenia (SARC-F ≥ 6 points) and muscle function decline (SARC-F ≥ 4 points) were 17.3% and 34.6%, respectively. The authors proposed the addition of calf circumference measurements to SARC-F to improve the instrument’s measurement accuracy.^18^ The EWGSOP2 establishes an overlap of muscle strength in relation to muscle mass as a primary parameter in the diagnosis of sarcopenia, as muscle strength is the most reliable measure of muscle function.^10^ Furthermore, it is associated with adverse health outcomes and facilitates the use of the diagnostic algorithm in clinical practice.^10,15,31^ Thus, the present study considered values ≥ 4 in the SARC-F as the cutoff point because of the improved accuracy in diagnosing muscle function in older Brazilian people and support from the scientific community.^10,14,15,17^ In addition, it is impossible to conduct anthropometric measurements due to pandemic-related restrictions.

Findings on sociodemographic differences between participants with and without RS were similar to those found in studies before the pandemic, whether in older patients with RS or with sarcopenia or on diagnostic parameters for sarcopenia.^18,25,26,27,31-35^ The difference in the presence of moderate to severe functional limitation between the groups was significant. After adjusted logistic regression, those with moderate to severe functional limitation were 19.05 times more likely to be at RS. Similar findings were reported by Rolland et al, with a sample of 3,025 French older adults (80.5 years).^33^ The authors found a lower functional performance in older adults at RS compared to the total sample and a significant association with reduced functional performance based on the gait speed and chair stand test results (OR: -0.04; CI 0.05–0.03 and OR: 13.1; CI 11.5–14.7). Longitudinal analyses with a 6-year follow-up confirmed the ability of SARC-F score ≥ 4 points (RS) to predict reduced functional performance.^31^

Our logistic regression analysis, adjusted for sociodemographic factors, showed a significant association of the presence of comorbidity with RS, corroborating previous studies.^25,33^ Given the context of the pandemic, the combination of psychobehavioral factors, such as stress, worse sleep quality, food routine, and mood, as well as medical treatment and functional rehabilitation discontinuation, increased physical inactivity and SB, which triggered an accelerated progression of established chronic diseases due to the greater active systemic pro-inflammatory profile and higher muscle catabolism drive. Thus, monitoring these factors in older adults is necessary during and after the pandemic, including sociodemographic factors and their specifications.^1,2,3,36^

Pain contributed to the highest RS among the participants in this study. Corroborating this study, Lustosa et al. investigated RS in 322 older Brazilian women complaining of non-specific acute lower back pain, and the results showed an association between pain intensity and poor mobility and balance.^37^ The authors pointed out that RS, if present in older women with lower back pain, can negatively influence functionality.^37^ Pain is multifactorial and subjective. Moreover, psychosocial factors are known to interfere with pain and its pro-inflammatory process, and social isolation predisposes to the development of chronic pain.^38^ Thus, pain in older people should not be neglected during and after the pandemic, and directions for non-pharmacological and pharmacological interventions should be considered.

A positive and significant association was observed between SB and RS, with a “dose-response” effect for a more extended period of SB, causing older adults with 10 h or more of SB per day to be 3.93 (CI 2.48–6.22; compared to < 4 hours) times more likely to be at RS. With a sample of 1,068 older adults (72.1 years), Tzeng et al. demonstrated that sitting for 7 hours or more per day was significantly associated with RS (OR: 1.98; CI 1.09–3.59).^39^ Smith et al. also investigated the relationship between SB and sarcopenia in 14,585 older adults from six low- and middle-income countries.^40^ The authors identified that regardless of the PA level and presence of comorbidities, 11 hours or more of SB increases RS by 2.14 times (CI 1.06–4.33; compared to < 4 hours), and each additional hour per day of SB was related to an increased risk of RS by 1.06 (CI 1.04–1.10).^40^ Thus, our results confirm that the more sedentary the lifestyle during the pandemic, the greater the probability of RS and possibly the worse is the health condition and muscle function prognosis.

It is known that physical inactivity and PA levels below the recommendations proposed by the World Health Organization (WHO) are more frequent in older adults,^3,41,42^ and sarcopenic individuals have lower PA levels than non-sarcopenic individuals.^25,34,43^ In the present study, there was a difference in walking (exercise, utilitarian, and total) between the two groups, reflecting the low PA level in participants with RS during the pandemic. Saraiva et al. found a reduction in the practice of regular PA (≥ 3 times/week) during the pandemic in 557 older Brazilian (80 ± 8 years), ranging from 42% active (pre-pandemic) to 26% (during the pandemic).^44^ Tzeng et al. showed that insufficiently active older adults had a 5.14 (CI 3.04–8.70) times higher RS.^39^ Thus, physical inactivity is a modifiable risk factor for sarcopenia, and physical exercise is the first-line treatment for this muscle disease.^2,15,32^

Our results showed lower life space mobility during the pandemic in the RS group. A similar and significant difference was found in a study published before the pandemic.^45^ In this study, the group without RS had lower average age, was more active, and presented with a lower percentage of comorbidity than the group with RS. Higher mobility rates are associated with better muscle function, functional and cognitive performance, and social support.^46^ This finding serves as a warning for this target population, given the prolonged course of the pandemic and the deleterious relationship between restriction of outdoor mobility and skeletal musculature.

Some limitations of this study must be considered. Snowball sampling was carried out on an online platform, differentiating our sample from the general community. The participants could have had access to the Internet and a higher level of education or social support as opposed to the older Brazilian population in general. Our findings were extrapolated to older adults with characteristics similar to those of our sample. In addition, the study had a cross-sectional design, making it impossible to identify causality in the analyzed relationships. However, this cross-sectional analysis aimed to identify and verify RS and its contributing factors in the Rede Remobilize (Wave 1) cohort and establish a baseline for future longitudinal studies on the impacts of the pandemic and RS in older individuals. To our knowledge, this is the first study to assess RS in a consistent sample of community-dwelling older adults in Brazil during the pandemic. Finally, this study encourages the use of SARC-F in monitoring older patients because it is a viable tool in clinical practice for screening for muscle function decline and RS, as it allows for the adequacy of future health care actions in favor of healthy aging.^19,47^

## CONCLUSIONS

Moderate to severe functional limitation, comorbidity, pain, longer period of SB, and reduced life space mobility significantly contributed to the RS in older Brazilian adults during the pandemic. Longitudinal studies monitoring functional trajectories and adverse health outcomes in older patients with RS during the pandemic should be encouraged to understand the associated modifiable factors and preventive actions against this critical muscle dysfunction.
